# Successful Cesarean Section for Dystocia due to Incomplete Cervical Dilation in a Doe: A Veterinary Case Report

**DOI:** 10.1002/ccr3.73028

**Published:** 2026-06-22

**Authors:** Chukwuka Elendu, Dependable C. Amaechi, Tochi C. Elendu, Emmanuel C. Amaechi, Ijeoma D. Elendu

**Affiliations:** ^1^ Federal University Teaching Hospital Owerri Nigeria; ^2^ Igbinedion University Okada Nigeria; ^3^ Imo State University Owerri Nigeria; ^4^ Madonna University Elele Nigeria; ^5^ Gregory University Uturu Nigeria

**Keywords:** cervical dystocia, cesarean section, goat obstetrics, parturition, small ruminants

## Abstract

Failure of cervical progression during prolonged labor should prompt early reassessment rather than repeated obstetric manipulation. In goats, persistent cervical rigidity despite ongoing uterine activity warrants timely escalation of care to avoid preventable complications in both the doe and kid, particularly in field and smallholder practice settings.

## 1 Introduction and Background

Dystocia is a common obstetric complication in goats and an important cause of maternal and neonatal morbidity and mortality, particularly in low‐resource production systems [1]. It may result from fetal factors, such as malpresentation or fetal oversize, or maternal factors, including uterine inertia, pelvic abnormalities, and failure of cervical dilation [2]. Incomplete cervical dilation (cervical dystocia) is an uncommon but serious maternal cause of obstructive labor that may not respond to medical or manual intervention [3].

Normal parturition requires progressive cervical softening and dilation, and failure of this process can result in prolonged labor, fetal compromise, and maternal complications if not promptly addressed [4, 5]. In such cases, cesarean section is often the definitive treatment, particularly when conservative measures are unsuccessful or fetal viability is at risk [5, 6]. This report describes the successful surgical management of cervical dystocia caused by incomplete cervical dilation in a doe and highlights the importance of timely recognition and intervention.

## 2 Case Description

A multiparous adult doe managed under a smallholder production system in a rural setting was presented to a veterinary practitioner with a history of prolonged labor characterized by repeated unsuccessful attempts at delivery despite sustained abdominal straining. According to the owner, the doe had been in active labor for approximately 14 h prior to presentation, during which time restlessness, frequent postural changes, and intermittent vocalization were observed. The animal had previously kidded without complications and had no known history of reproductive disorders, trauma, systemic illness, or exposure to exogenous hormones. Gestation was estimated to be at term based on breeding records, progressive abdominal enlargement, udder development, and relaxation of the pelvic ligaments, and no abnormalities had been noted during the antenatal period.

**FIGURE 1:**
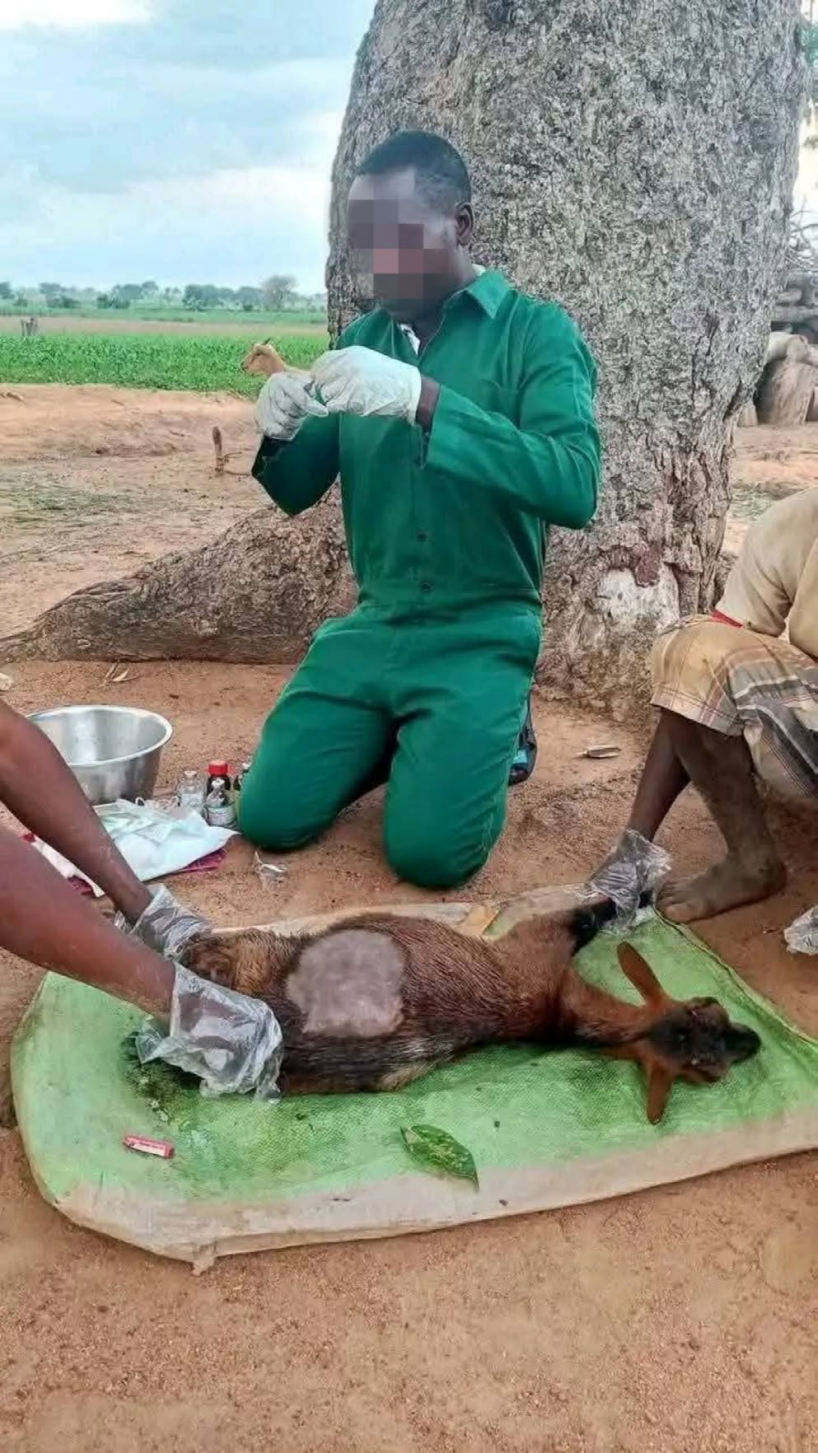
Doe in lateral recumbency during prolonged labor showing absence of externally visible fetal parts at presentation.

On initial examination, the doe was alert but visibly exhausted, with reduced responsiveness to external stimuli and signs of discomfort. Vital parameters revealed mild tachycardia and tachypnea consistent with prolonged labor stress, while rectal temperature remained within the normal physiological range. The mucous membranes were pink and moist, and capillary refill time was within normal limits, indicating preserved peripheral perfusion. Abdominal palpation demonstrated intermittent uterine contractions; however, these appeared weak and poorly coordinated. Vulvar examination revealed vulvar edema with serosanguinous discharge; however, no fetal parts were visible externally (Figure 1).

A careful vaginal examination was performed using appropriate restraint and lubrication. The vaginal canal was patent and adequately lubricated; however, the cervix was firm, thickened, and only minimally dilated, admitting no more than one to two fingers despite the prolonged duration of labor. No fetal extremities, head, or fetal membranes could be palpated through the cervical opening, and gentle attempts at manual dilation were unsuccessful. The cervix failed to respond to sustained digital pressure, and no evidence of cervical tearing, torsion, or vaginal obstruction was identified. Based on these findings, the clinical presentation was consistent with dystocia due to incomplete cervical dilation.

## 3 Differential Diagnosis, Treatment Plan, and Follow‐Up

Cervical dystocia was considered the most likely diagnosis; however, other causes of dystocia in goats, including fetal oversize, malpresentation, malposition, fetal abnormalities, uterine inertia, uterine torsion, cervical torsion, pelvic narrowing, and vaginal abnormalities, were also considered [3, 7, 8]. The presence of persistent straining, ongoing uterine contractions, a firm minimally dilated cervix, and the absence of detectable vaginal or pelvic abnormalities supported incomplete cervical dilation as the primary cause of the obstructive labor. Although uterine torsion may be difficult to diagnose before surgical exploration, no evidence of uterine torsion was identified during cesarean section.

Management focused on resolving the obstructive labor while minimizing maternal and fetal risk; persistent cervical non‐dilation made conservative obstetric interventions unlikely to be successful. Oxytocin was avoided, as augmentation of uterine contractions in the presence of a non‐dilated cervix may increase the risk of uterine rupture, cervical trauma, and fetal compromise [9]. Prostaglandin therapy was also not pursued because of its limited and unpredictable efficacy in established cervical non‐dilation during active labor [10]. Given the prolonged labor, failure of cervical response to manual manipulation, and concern for maternal exhaustion and fetal distress, an emergency cesarean section was selected as the definitive treatment.

**FIGURE 2:**
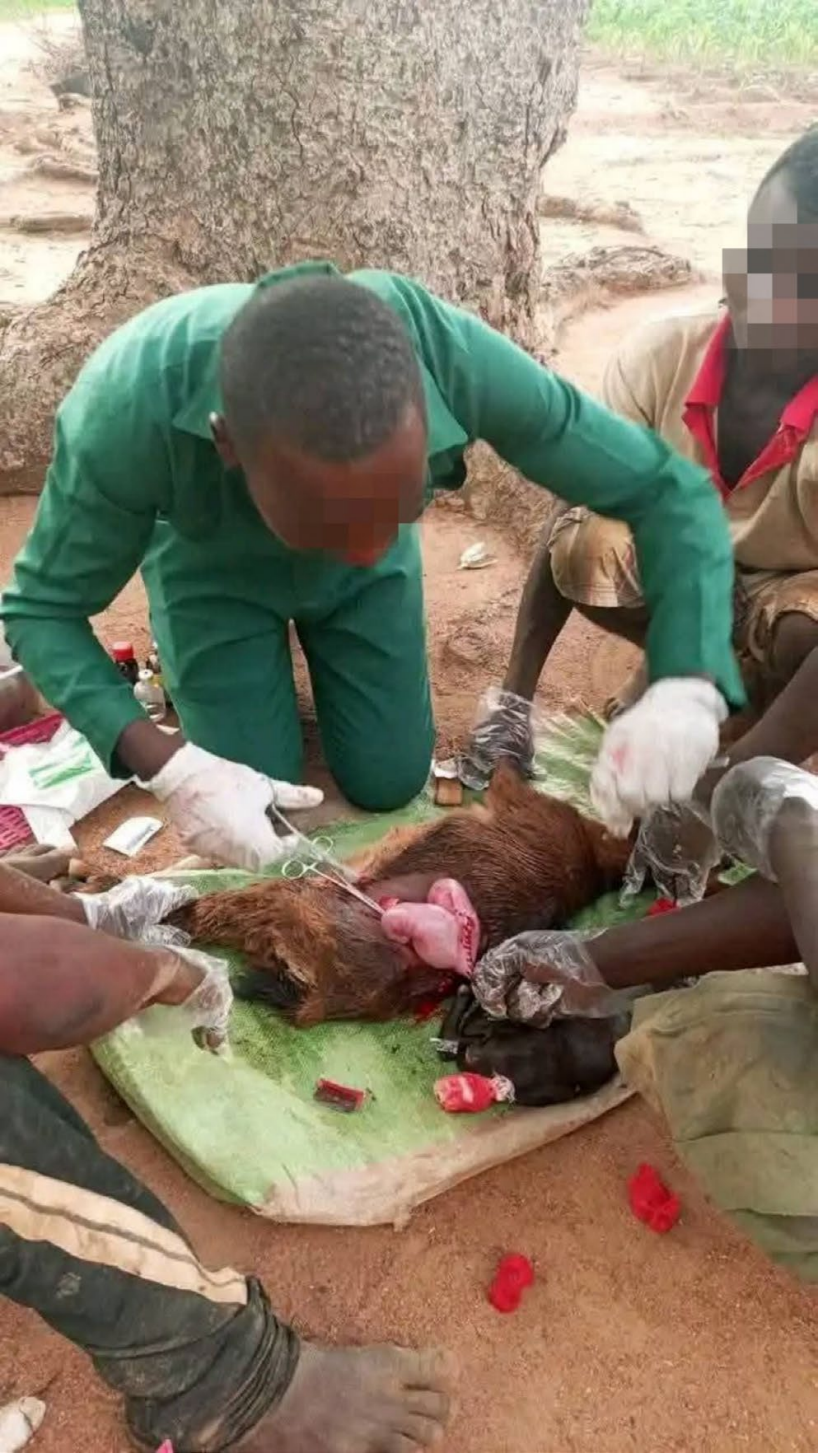
Intraoperative exposure of the gravid uterus following flank laparotomy in a doe with dystocia due to incomplete cervical dilation.

**FIGURE 3:**
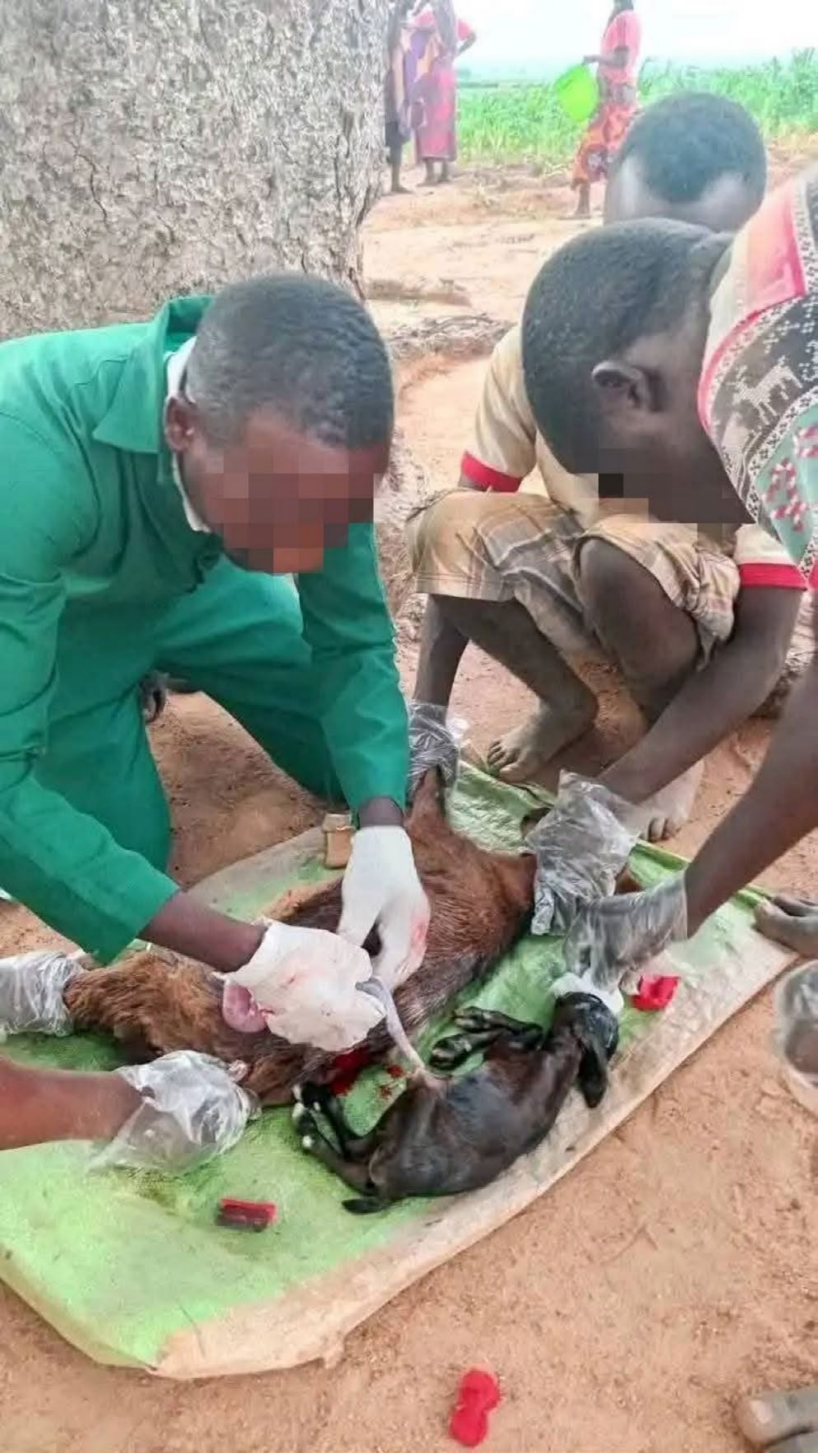
Delivery of a live kid through a uterine incision during cesarean section.

The procedure was performed through a standard left paralumbar flank approach under local infiltration anesthesia using 2% lidocaine hydrochloride (6 mg/kg) [11]. The gravid uterus was exteriorized and incised, allowing delivery of a live kid (Figures 2 and 3). The uterus was examined for additional fetuses and structural abnormalities before routine closure and replacement into the abdominal cavity. The abdominal wall and skin were closed in layers, and the procedure was completed without intraoperative complications. The placenta was subsequently expelled without complication, with no evidence of retained fetal membranes.

**FIGURE 4:**
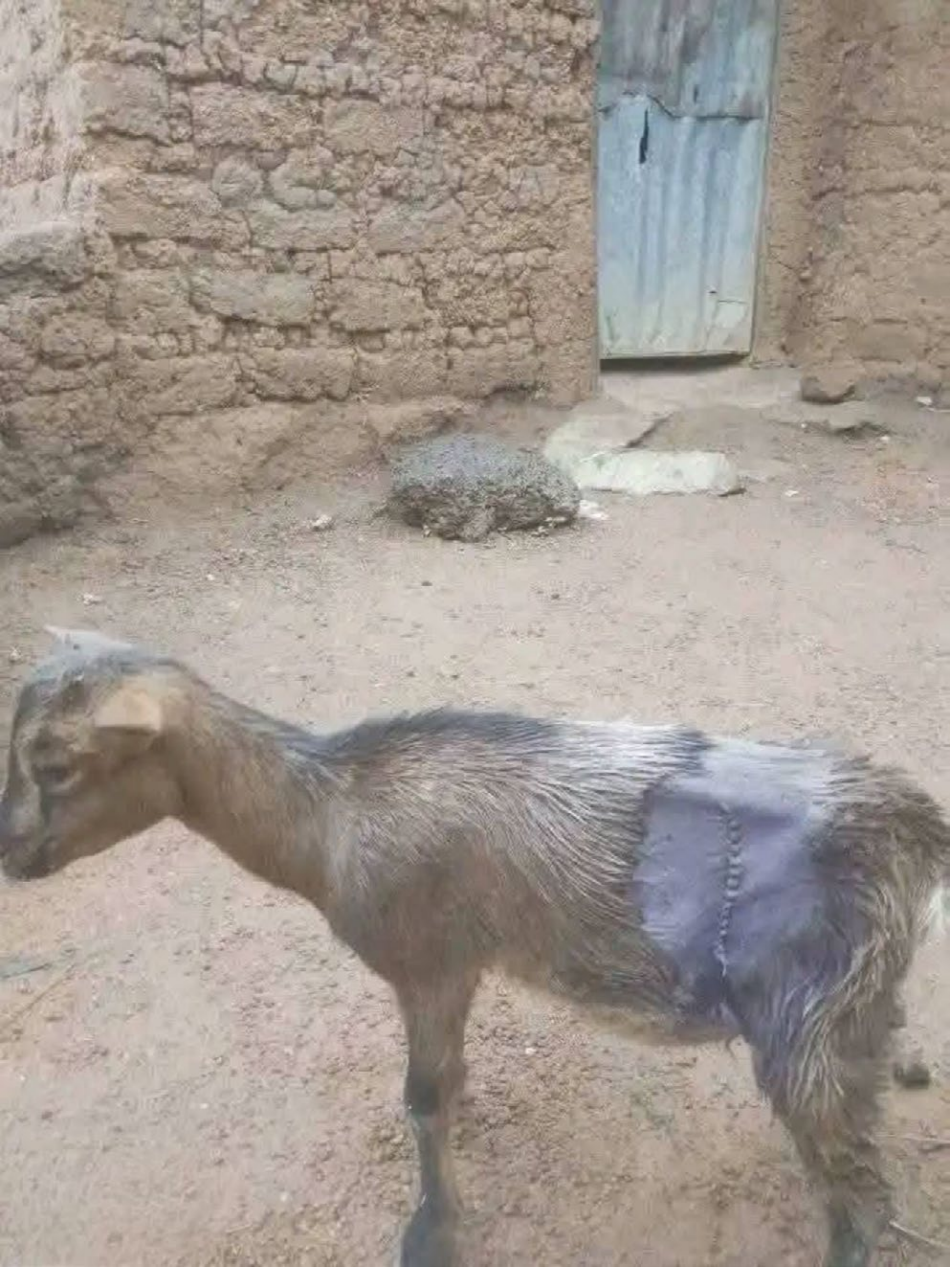
Postoperative appearance of the doe showing the surgical site following cesarean section.

Postoperatively, the doe received oxytetracycline (20 mg/kg IM) and meloxicam (0.5 mg/kg SC) once daily for 3 days and was monitored for complications [12]. She regained normal posture within hours of surgery and resumed voluntary feed intake within 24 h, with normal urination and defecation observed. No abnormal vaginal discharge or signs of systemic illness were noted during recovery (Figure 4).

The kid demonstrated spontaneous respiration, normal reflexes, and adequate vigor immediately after delivery. Assisted nursing was provided to ensure adequate colostrum intake [13], and the kid remained active and responsive, with no congenital abnormalities identified on physical examination.

Follow‐up evaluation several days after surgery revealed satisfactory healing of the surgical incision without wound dehiscence, infection, or herniation. The doe exhibited normal maternal behavior and adequate milk production, and the kid continued to thrive with appropriate weight gain and activity levels.

## 4 Discussion

Dystocia due to incomplete cervical dilation is an uncommon but clinically important cause of obstructive labor in goats. Normal cervical dilation during parturition depends on coordinated hormonal, biochemical, and mechanical processes. Disruption of these mechanisms may result in failure of cervical softening and dilation despite ongoing uterine activity, a condition associated with endocrine disturbances, altered prostaglandin metabolism, collagen dysregulation, or idiopathic cervical rigidity [3, 4]. Unlike uterine inertia, true cervical non‐dilation represents a mechanical obstruction that is often refractory to medical therapy and manual manipulation [5, 6]. However, denaverine hydrochloride, a spasmolytic agent licensed for use in cattle, has been used off‐label in small ruminants to promote cervical relaxation and facilitate cervical dilation in cases of incomplete cervical dilation. Although not used in the present case, its reported benefits are largely based on anecdotal experience and field use, while published evidence regarding its efficacy, safety, and availability in goats remains limited [14, 15].

Although cervical dystocia is less frequently reported in goats than in cattle, it remains a recognized cause of maternal dystocia in small ruminants [1, 2, 6]. The absence of palpable fetal parts on vaginal examination may occur in cases of cervical non‐dilation because access to the uterine contents is restricted [7]. This feature may complicate differentiation from other causes of obstructive labor and highlights the importance of thorough obstetrical assessment.

Cesarean section remains the treatment of choice for cervical dystocia when conservative measures are unsuccessful or contraindicated [1, 5, 6]. The favorable outcomes observed in both the doe and kid are consistent with previous reports demonstrating satisfactory postoperative recovery following timely surgical intervention [11, 12]. Although a live kid was delivered in the present case, prolonged dystocia may occasionally be complicated by fetal mummification or putrefaction. In such situations, management and prognosis may be influenced by the degree of fetal decomposition, uterine contamination, and the maternal condition at presentation [5, 6].

The precise etiology of cervical non‐dilation often remains undetermined, although factors such as primiparity, advanced maternal age, previous cervical trauma, and endocrine disturbances have been proposed [1, 3]. Breed‐related differences in cervical compliance and parturition dynamics have also been suggested in goats, although supporting evidence remains limited [6]. Further studies are needed to clarify the pathophysiology and risk factors associated with cervical dystocia and to guide preventive and therapeutic strategies.

## 5 Concluding Remarks

Incomplete cervical dilation is an important cause of dystocia in goats and may not respond to conservative obstetric management. In the present case, cesarean section resulted in favorable outcomes for both the doe and kid. Awareness of cervical dystocia as a cause of prolonged, non‐progressive labor may facilitate appropriate case management and improve clinical outcomes.

## Author Contributions

**Chukwuka Elendu:** conceptualization, investigation, project administration, supervision, validation, visualization, writing – original draft. **Dependable C. Amaechi:** data curation, methodology, writing – review and editing. **Tochi C. Elendu:** data curation, writing – review and editing. **Emmanuel C. Amaechi:** funding acquisition, validation, writing – review and editing. **Ijeoma D. Elendu:** visualization, writing – review and editing.

## Funding

The authors have nothing to report.

## Ethics Statement

Ethical approval was not required in accordance with institutional policy.

## Consent

Written informed consent was obtained from the animal owner for publication of this report and accompanying images.

## Conflicts of Interest

The authors declare no conflicts of interest.

## Data Availability

All relevant data supporting the findings of this report are included within the article.
